# Lipid dysfunction and adrenomedullin expression in omental versus subcutaneous adipose tissues in diabetic pregnancies

**DOI:** 10.1371/journal.pone.0265419

**Published:** 2022-04-07

**Authors:** Yuanlin Dong, Ancizar Betancourt, Michael A. Belfort, Chandrasekhar Yallampalli

**Affiliations:** Department of Obstetrics and Gynecology, Baylor College of Medicine/Texas Children’s Hospital, Houston, Texas, United States of America; Loma Linda University School of Medicine, UNITED STATES

## Abstract

Gestational diabetes mellitus (GDM) is one of the most common complications of pregnancy but the underlying mechanism remains obscure. The aims of this study are to examine if omental adipose tissue (OMAT) and subcutaneous AT (SCAT) differentially express proinflammatory and lipid metabolic adipokines, and if so, whether their regional differences have implications on lipid metabolism in GDM. Paired samples of OMAT and SCAT were excised from pregnant women in scheduled Cesarean sections with non-obese (NOBS), obese (OBS) and GDM. The results showed that the mRNA of monocyte chemoattractant protein (MCP)-1, macrophage marker CD68, and cytokines IL-6, IL-8, and TNF-α are increased in OMAT from GDM women compared to that in NOBS and OBS women (P<0.05). Glucose and TNF-α dose-dependently enhanced ADM and its receptor components CRLR and RAMPs in human adipocytes. Immunofluorescence showed that ADM and its receptor components are higher in OMAT from GDM women compared to non-GDM women. Further, basal lipolysis was greater in OMAT than in SCAT and ADM stimulates further glycerol release in OMAT, but not in SCAT, and these increases are reduced by ADM antagonist, ADM22-52. We therefore conclude that elevated ADM and its receptor expressions by OMAT, but not by SCAT appear to contribute to the lipid dysregulation in GDM women, and manipulation of ADM may represent one of the novel approaches in minimizing the risk of GDM-related fetal overgrowth.

## Introduction

Gestational diabetes mellitus (GDM) is a public health concern which affects up to 18% of pregnancies [1]. The incidence of GDM-associated complications are increased in both the mother and the fetus, including gestational hypertension and preeclampsia in the mother, and hyperglycemia and macrosomia in the infant [2]. Although extensive investigations have been made in the past, the underlying pathophysiology of GDM remain obscure. Evidence from clinical and experimental studies have suggested that maternal adipose tissue dysfunction, characterized by changes in adipokines and lipid metaboliosm [3], adversely affects the quality and quantity of lipids transferred to the fetus altering the fetal adiposity and development. However, the underlying mechanism for the impaired lipid metabolism in diabetic pregnancy remains unclear.

Human adipose tissue is one of the heterogeneous organs with both metabolic and endocrine function. Tissue site differences in the breakdown of intracellular triglyceride stores have been reported [4]. It has been shown that catecholamines stimulate lipolysis more markedly in the omental adipose tissue (OMAT) than in the subcutaneous adipose tissue (SCAT), and insulin is less antilipolytic in the OMAT compared with SCAT [5]. The OMAT, but not the SCAT, has a direct access to the liver through the portal vein, and there is excess free fatty acid release from the visceral AT in central obesity [6]. The latter might interfere with liver metabolism and contributes to the development of glucose intolerance, hyperinsulinemia, and hypertriglyceridemia, but the involvement of OMAT and SCAT in lipid dysregulation in diabetic pregnancy is still unclear.

Adipocytes take up glucose and store energy in the form of triglycerides under the regulation of insulin, which are subsequently broken down into free fatty acids (FFAs) and glycerol when the energy is demanded. In addition to the fat storage, adipocytes also synthesize and secrete a variety of bioactive adipokines, including proinflammatory and lipid metabolic molecules retinol-binding protein-4 (RBP-4), toll-like receptor-4 (TLR-4), carbohydrate responsive element-binding protein (ChREBP), carboxyl ester lipase (CEL), monocyte chemoattractant protein (MCP)-1, and adrenomedullin (ADM) [7,8]. ADM is a potent vasodilator peptide originally isolated from human pheochromocytoma [9], and its effects are primarily mediated through calcitonin receptor-like receptor (CRLR). CRLR interacts with receptor activity-modifying proteins (RAMPs), among which RAMP2 and RAMP3 carry CRLR from the endoplasmic reticulum to the cellular membrane to confer high affinity for ADM [10]. ADM is expressed by a variety of cell types, and functions in an autocrine/paracrine fashion [11] exerting its effects on cell growth [12], inflammation [9], hormone secretion [13], pregnancy related vascular adaptations [14] and fetal growth [15]. Recent studies show that ADM and its receptors are expressed in rat adipose tissue [16], administration of ADM induces hyperglycemia, which can be reversed by an ADM neutralizing antibody [17]. In humans, plasma ADM concentrations are elevated in obese individuals [18] and patients with T2DM [19]. Our previous studies have demonstrated that both circulating ADM and ADM expression in OMAT were increased in GDM patients [20,21], indicating that ADM may be involved in the pathogenesis of insulin resistance in GDM as observed in diabetes. However, the adipose tissue-site related differences in ADM and its receptors expression in normal pregnancies and GDM remain unknown. Therefore, the present study was designed to determine if OMAT and SCAT differentially express proinflammatory and lipid metabolic adipokines, including ADM system, and if so, whether the site-specific differences in their expressions have implications on lipid homeostasis in gestational diabetes. In addition, we assessed if these changes are dependent up on the body mass index (BMI).

## Materials and methods

### Subjects

This study was approved by Baylor College of Medicine Institutional Review Board (IRB # H28527), and was conducted according to Declaration of Helsinki Principles. Informed consents were obtained from all participating subjects admitted to the Pavilions for Women at Texas Children’s Hospital (TCH), who are scheduled for elective non-laboring Cesarean section for the delivery at term and enrolled in the study. BMI was calculated based on the recorded height and the weight, either measured at the first visit during early pregnancy or self-reported as pre-pregnancy weight. Paired SCAT and OMAT was obtained from women with non-obese (NOBS, BMI<30 kg/m^2^, n = 8), obese (OBS, BMI>30, n = 8) and GDM (n = 7) during Caesarian sections between October 2017 and March 2020. All women were screened for GDM, and patients in other groups had negative screening results. The 1-hour glucose screen cutoff for GDM is 140 mg/dl. The 3-hour glucose tolerance test (GTT) values are fasting > 95 mg/dl; 1 h > 180 mg/dl; 2 h > 155 mg/dl; 3 h >140 mg/dl. Pregnant patients were excluded from participating in the study if they had preexisting diabetes, fetal anomalies, multifetal pregnancy, hypertension, preeclampsia, immunosuppressive treatment, or clinical evidence of maternal or fetal infection. The relevant clinical details of the subjects were shown in Table 1.

**Table 1:** Patient characteristics.

Groups	NOBS	OBS	GDM	p
Number	8	8	7	
Ethnicity (Non-Hispanic/Hispanic)	6/2	6/2	¾	
Maternal age (Years)	32.6 +/- 1.4	30.9 +/- 1.1	32.3 +/- 2.2	0.588
Gestational Age (Weeks)	38.6 +/- 0.5	38.6 +/- 0.2	38.1 +/- 0.4	0.384
Fetal Gender (Male/Female)	1/7	3/5	¾	
Birth Weight (Grams)	3327+/-103	3249+/-129	3671+/-103*	0.046
BMI (kg/m^2^)	27.3 +/- 0.9	37.9 +/- 0.9**	36.7 +/- 1.8**	0.0006
Fasting blood glucose (mg/ml)	94.3 +/- 5.3	92.6 +/- 4.9	86.9 +/- 6.7	0.5321
Diet CTL/ Insulin	0/0	0/0	2/5	

### Quantitative Real-Time PCR

Total RNA was isolated from OMAT and SCAT using TRIzol (Life Technologies, Grand Island, NY) and RT was performed as previously described [22]. Quantitative Real-time-PCR was performed using Taq universal SYBR Green Supermix (Bio-Rad). The primers used for ADM (Cat #: Hs00181605), CRLR (Cat #: Hs00173787), RAMP2 (Cat #: Hs00359352), and RAMP3 (Cat #: Hs00389130) are commercially available from Life Technologies, (Grand Island, NY). PCR primers used for amplification of lipid metabolism and proinflammatory molecules were purchased from Integrated DNA Technologies (IDT) and the primer sequences were list in Table 2. Amplification of GAPDH served as an endogenous control. PCR conditions for SYBR Green gene expression were 10 min at 95°C for 1 cycle, then 15 sec at 94°C, 30 sec at 60°C and 15 sec at 72°C for 39 cycles. All experiments were performed in triplicate. The average CT value was used to calculate the results using the 2–ΔΔCT method, and expressed in fold increase/decrease of the gene of interest.

**Table 2:** Primer sequences.

	Forward	Reverse
RBP-4	5’TTCGATAAGGCTCGCTTCTC3’	5’CGATGTTGTCCTGCAGAAAGAG3’
TLR-4	5’GCATCTGGCTGGGACTCT3’	5’TAGTCCATGCATTGGTAGG3’
ChREBP	5’ AGCGGATTCCAGGTGAGG3’	5’TTGTTCAGGCGGATCTTGTC3’
CEL	5’AGAAGGTGGGTTCGTGGAAG3’	5’GAAGGTGACCACGATGACGT3’
MCP-1	5’CCCCAGTCACCTGCTGTTAT3’	5’AGGTGACTGGGGCATTGATT3’
CD68	5’ GCTACATGGCGGTGGAGTACAA3’	5’ATGATGAGAGGCAGCAAGATGG3’
IL-6	5’AAATGCCAGCCTGCTGACGAAG3’	5’AACAACAATCTGAGGTGCCCATGCTAC3’
IL-8	5’TTGGCAGCCTTCCTGATTTC3’	5’AACTTCTCCACAACCCTCTG3’
TNF-α	5’TCAGGATCATCTTCTCGAACC3’	5’GAGTCCTTCTCACATTGTCTC3’
GAPDH	5’GGTCTCCTCTGACTTCAACA3’	5’AGCCAAATTCGTTGTCATAC3’

### Human pre-adipocyte culture

Primary human pre-adipocytes (ATCC PCS-210-010) were differentiated into mature adipocytes in wells of 24-well-plates containing adipocyte differentiation medium (Cell Applications, Inc. San Diego, CA) in a 5% CO_2_ atmosphere at 37° C. According to the product sheet from ATCC, these human pre-adipocytes are derived from de-differentiated mature adipocytes and provide an ideal culture model for the study of diabetes, obesity, metabolism, insulin sensitivity and adipose biology. These cells can be expanded in an undifferentiated state for future differentiation to mature adipocytes and show higher efficiency of adipogenesis compared to mesenchymal stem cells. In this study, the basic concentration of glucose in adipocyte differentiation medium from Cell Applications Inc. is 1.5mg/ml. The cells were then treated with increasing dose of glucose (2.0–3.5 mg/ml, Sigma-Aldrich, St. Louis, MO), or TNF-α (0–0.5 ng/ml, Sigma-Aldrich) for 24 hours. Total RNA was isolated from the cells using TRIzol (Life Technologies, Grand Island, NY) and RT was performed for further Quantitative Real-time-PCR analysis.

### Immunofluorescent imaging analysis

Optimal cutting temperature (OCT) medium embedded SCAT and OMAT were cut at 5- to 7-μm thickness and mounted on gelatin-coated slides as previously described [23]. The sections were fixed with methanol and acetone mixture (1:1), and then the first and second primary antibodies, rabbit anti-perilipin monoclonal antibody (the marker for adipocyte, Cell signaling, Beverly, MA), goat polyclonal ADM and CRLR antibody (Santa Cruz Biotechnology Inc., Dallas, TX), and rabbit RAMP2 and RAMP3 polyclonal antibodies [24] were applied at 1:1000 dilution, followed by either goat anti-mouse IgG-FITC (Southern Biotech, Birmingham, AL), or donkey anti-goat Alexa Flour 594/anti-rabbit IgG TRITC (Life Technology, Grand Island, NY). The slides were then mounted with mounting-medium containing 4′, 6-diamidino-2-phenylindole (DAPI; Vector Laboratories Inc., Burlingame, CA) and viewed under an Olympus BX51 microscope. The intensity of the immunofluorescence was measured by using CellSence software (Olympus Scientific, Walthan MA, USA), and the relative densities of the immunofluorescence to the number of nuclei were calculated and compared between groups.

### Adipose tissue explant culture and glycerol measurements

OMAT and SCAT were finely diced and transferred to wells of 24-well plates containing DMEM (Gibco, Life technology, Gaithersburg, MD) and cultured in a humidified atmosphere of 21% O_2_ and 5% CO_2_ at 37°C for 1 hour [25]. After refreshing the medium, the tissues were incubated in the presence or absence of ADM with or without ADM22-52 (American Peptide Co., Inc. Sunnyvale, CA) for 24 hours. ADM22-52 was added 30 minutes before ADM treatments. The glycerol release into cell culture medium was assessed by using Free Glycerol Reagent (Sigma Aldrich, St. Louis, MO) as manufacture’s instruction. The absorbance at A540 were recorded by using Spectrophotometer CLARIO STAR (BMG Labtech, Inc., Cary, NC).

### Statistics

All data were presented as mean ± SEM. Data were calculated and analyzed by GraphPad Prism (La Jolla, CA). Repeated measures ANOVA (treatment and time as factors) with a Bonferroni post hoc test were used for comparisons between groups. mRNA and protein expression were compared between control and treatment groups using unpaired Student *t* test. Statistical significance was defined as *p*<0.05.

## Results

The demographic data of participants in this study were summarized in Table 1. There were 6 non-Hispanic and 2 Hispanic women in both NOBS and OBS group, 3 non-Hispanic and 4 Hispanic women in GDM group. There were no significant differences in maternal age, gestational age, and fasting blood glucose between groups. Infant birth weight in GDM group was much higher than that in NOBS and OBS groups (P = 0.046), but no significant differences were noted between NOBS and OBS group. As expected, the pre-pregnancy/early pregnancy BMI in OBS and GDM were significantly higher than NOBS group (P = 0.0006), but there were no significant differences in BMI between OBS and GDM groups. In addition, 5 out of 7 women with GDM were prescribed insulin and 2 out of 7 under dietary management.

### mRNA of RBP4, TLR-4, ChREBP and CEL expression in adipose tissues

**Fig 1:**
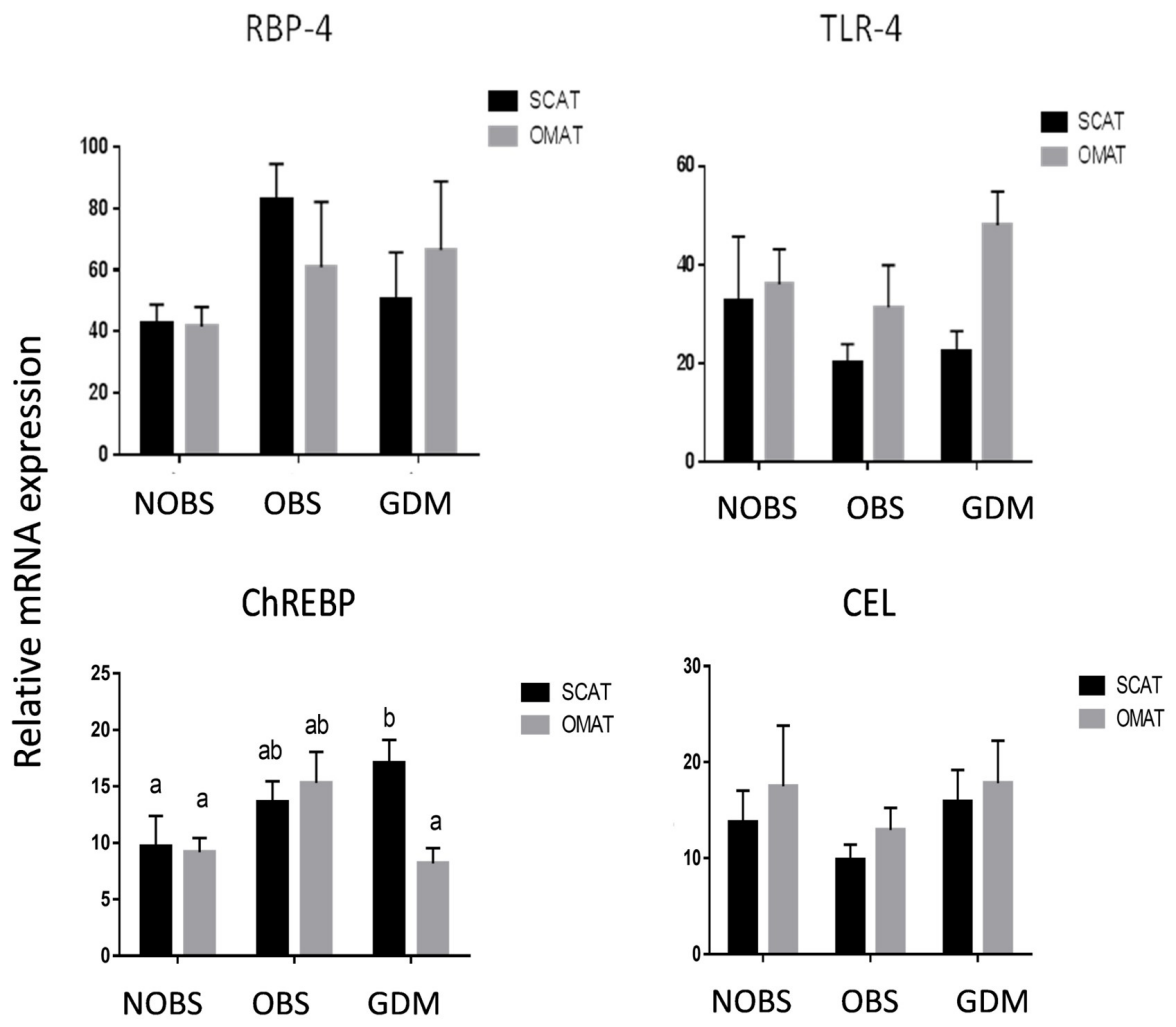
mRNA expression of RBP-4, TLR-4, ChREBP, and CEL in human adipose tissues. mRNA expression of retinol binding protein 4 (RBP-4), toll-like receptor 4 (TLR-4), carbohydrate responsive element binding protein (ChREBP), and carboxyl ester lipase (CEL) in SCAT and OMAT from pregnant women with NOBS (n = 8), OBS (n = 8), and GDM (n = 7). mRNA were determined by using Real-Time PCR with specific primers. Data are displayed as mean+/- SEM. Different letters on the top of the bars (a and b) indicate significant differences between groups (p<0.05).

Real-Time q-PCR were performed to evaluate differences in several adipokine mRNA levels between SCAT and OMAT from women with NOBS, OBS and GDM. As shown in Fig 1, adipose tissues express mRNA for proinflammatory molecules of RBP4 and TLR-4, and adipogenic molecules of ChREBP and CEL. Relative mRNA expression for adipogenic molecule ChREBP is higher in SCAT but not in OMAT from GDM compared to both the AT sites in NOBS women (P<0.05). The mRNA for all other molecules were not different between SCAT and OMAT and among NOBS, OBS, and GDM patients (P>0.05).

### Proinflammatory adipokines are increased in OMAT in GDM women

**Fig 2:**
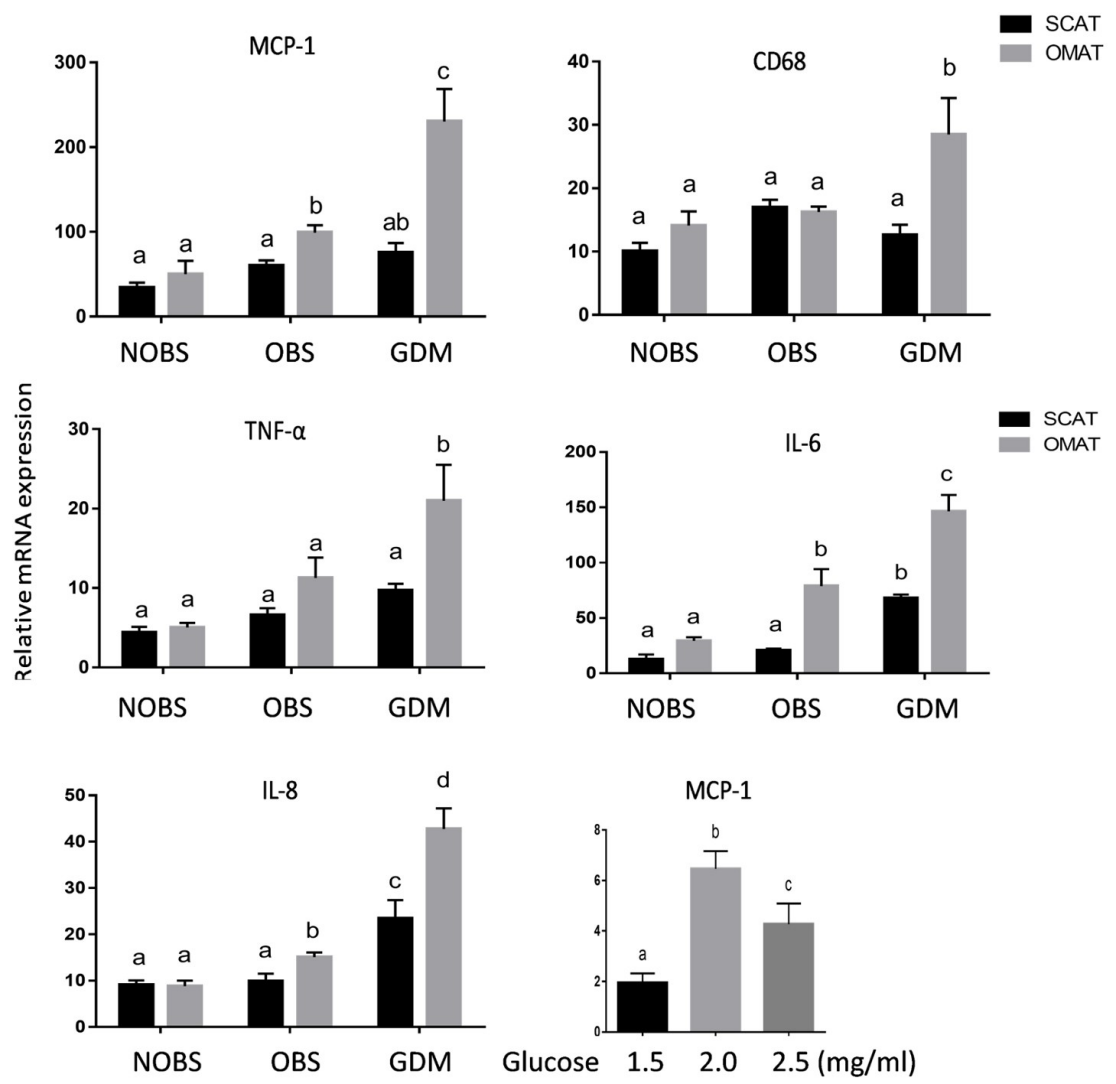
mRNA expression of proinflammatory adipokines in human adipose tissues. mRNA expression of monocyte chemoattractant protein 1 (MCP-1), macrophage maker CD68, TNF-α, IL-6, and IL-8 in SCATand OMAT from pregnant women with NOBS (n = 8), OBS (n = 8), and GDM (n = 7). mRNA were determined by using Real-Time PCR with specific primers. MCP-1 mRNA was also determined in human adipocytes treated with increasing doses of glucose for 24 hours (n = 6). Data are displayed as mean+/- SEM. Different letters on the top of the bars (a, b, c, and d) indicate significant differences between groups (p<0.05).

As shown in Fig 2, the mRNA of MCP-1, a monocyte chemoattractant protein, was substantially greater in OMAT but not in SCAT from GDM when compared to non-GDM groups. In addition, macrophage marker CD68, and cytokines IL-6, IL-8, and TNF-α were also significantly increased in OMAT, but not in SCAT from GDM women compared to that in NOBS and OBS women (P<0.05), implying the proinflammatory status of OMAT in GDM. Although, IL-6 mRNA was elevated in OMAT in OBS compared to NOBS women, the magnitude of this increase is smaller compared to that in GDM women. Furthermore, the MCP-1 mRNA expression in human adipocytes was stimulated by glucose treatment, indicating that enhanced MCP-1 expression in OMAT from GDM may result from hyperglycemia.

### Glucose and TNF-α enhances ADM and its receptor component mRNA in human adipocytes

**Fig 3:**
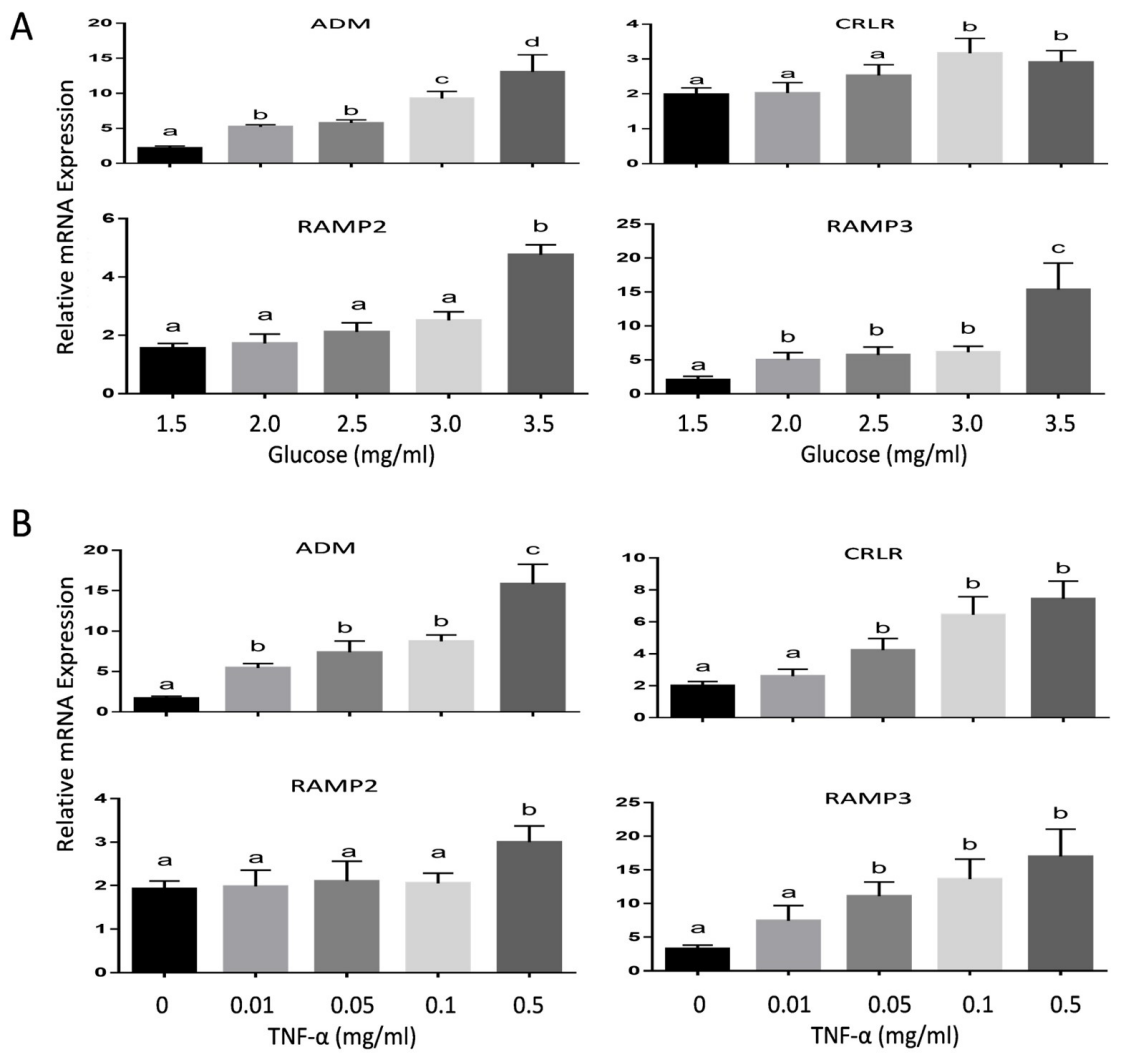
Regulation of mRNA for ADM and its receptors by glucose and TNF-α in human adipocytes. Muture adipocyts were incubated with increasing doses of glucose or TNF-α for 24 hours, and the mRNA expression for ADM, CRLR, RAMP2, and RAMP3 were determined by using Real-time qPCR with specific primers. Data are presented as mean +/- SEM (n = 6). Different latters on the top of the bars (a, b, and c) indicate significant differences between groups (p<0.01).

To determine the impact of hyperglycemia on ADM and its receptors in adipose tissue, we treated the human adipocytes with increasing doses of glucose. As shown in Fig 3A, increases in glucose concentrations from 1.5 mg/ml (the basic levels of glucose in this special adipocyte culture medium) to 3.5 mg/ml resulted in a dose-dependent increases in ADM mRNA expression (p<0.05). The mRNA expression for CRLR, RAMP2 and RAMP3 were also significantly increased when treated with increasing doses of glucose. Further, treatment of adipocytes with TNF-α from 0.01 ng/ml to 0.5ng/ml dose-dependently stimulates ADM mRNA expression (p<0.05) (Fig 3B). In addition, TNF-α elevated mRNA expression for CRLR, RAMP2, and RAMP3 in adipocytes (p<0.05) in a dose-dependent manner. Taking together, these results indicated that increased glucose and cytokines in GDM may contribute, at least in part, to the enhanced adipose tissue ADM and its receptor expression.

### GDM is associated with increased ADM and its receptor expression in OMAT but not in SCAT

**Fig 4:**
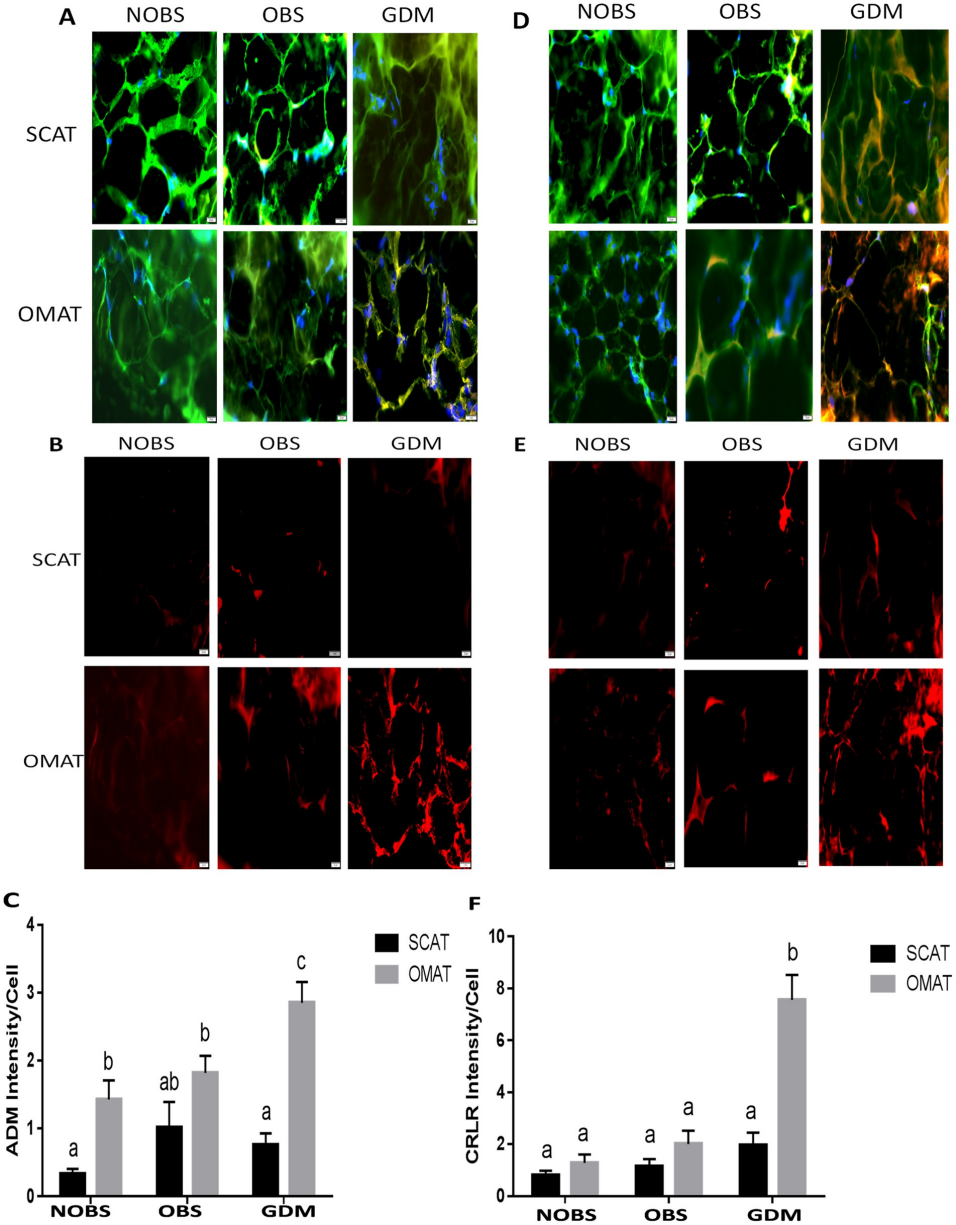
The immunofluorescence of ADM and CRLR in SCAT and OMAT. Presence of ADM in merged images (A, red) and non-merged images (B, red). and CRLR in merged images (D, red) and non-merged images (E, red) in the adipocytes (green) of SCAT and OMAT from NOBS (n = 8), OBS (n = 8), and GDM (n = 7) women were visualized by using specific antibodies. The relative densities of ADM (C) and CRLR (F) to the number of nuclei (blue) were compared between groups. Original magnification, x 400. Data are displayed as mean +/- SEM. Different letters on the top of the bars (a, b, and c) indicate significant differences (P<0.01).

**Fig 5:**
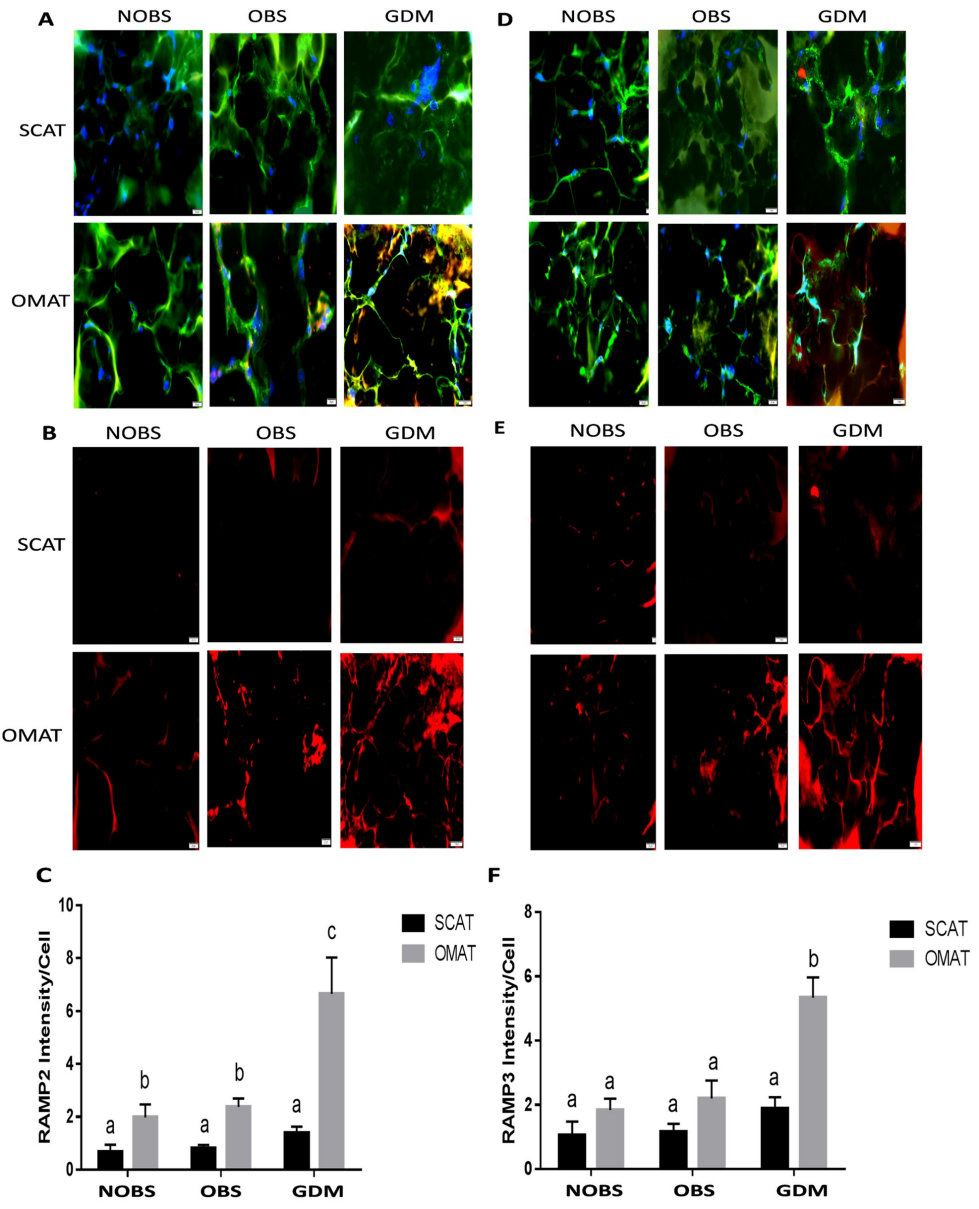
The immunofluorescence of RAMP2 and RAMP3 in SCAT and OMAT. Presence of RAMP2 in merged images (A, red) and non-merged images (B, red), and RAMP3 in merged images (D, red) and non-merged images (E, red) in the adipocytes (green) of SCAT/OMAT from NOBS (n = 8), OBS (n = 8), and GDM (n = 7) women were visualized by using specific antibodies. The relative densities of RAMP2 (C) and RAMP3 (F) to the number of nuclei (blue) were compared between groups. Original magnification, x 400. Data are displayed as mean +/- SEM. Different letters on the top of the bars (a, b, and c) indicate significant differences (P<0.01).

Immunofluorescent staining was performed to assess the protein expression of ADM and its receptor components in adipose tissues. As shown in Figs 4 and 5, ADM, and its receptor components CRLR, RAMP2 and RAMP3 were expressed in adipocytes from SCAT and OMAT segments tested. The intensity analysis showed that ADM protein was higher in OMAT compared with SCAT in both NOBS and GDM women, and OMAT from GDM subjects displayed highest ADM expression compared with either NOBS or OBS women. Further, the receptor components CRLR, RAMP2 and RAMP3, were also significantly higher in OMAT, but not in SCAT from GDM compared to OBS and NOBS women (P<0.05). The RAMP2 levels in OMAT were higher than SCAT in both NOBS and OBS groups. However, no significant differences were observed in ADM and its receptor components between non-GDM groups, irrespective of the BMI.

### Basal glycerol release was higher in OMAT from GDM women and ADM stimulates glycerol release in OMAT, but not in SCAT

**Fig 6:**
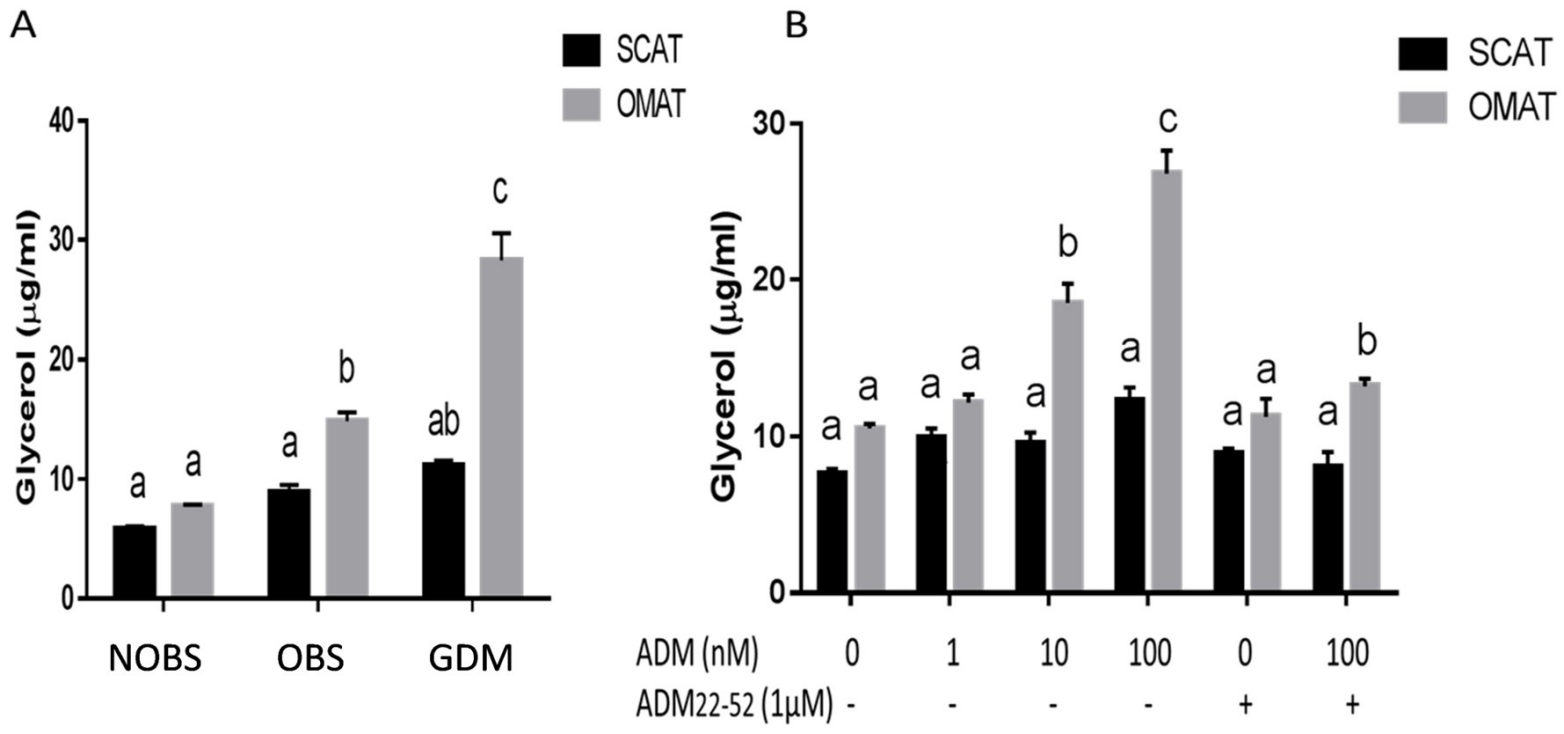
Basal and ADM-stimulated glycerol release by human adipose tissues. A) Basal glycerol release by SCAT and OMAT from NOBS (n = 8), OBS (n = 8) and GDM (n = 7) women after 24 hours explant culture. B) Effects of ADM on glycerol release by SCAT and OMAT from NOBS women (n = 8) in the presence or absence of ADM22-52 for 24 hours. ADM22-52 was added 30 minutes before ADM treatments. Data are displayed as mean+/-SEM. Different letters on the top of the bars (a, b, and c) indicate significant differences between groups (P<0.01).

To examine the lipid metabolic status in the patients recruited, we determined the basal glycerol release in adipose tissue explants in culture. As shown in Fig 6A, the basal glycerol release by OMAT was higher than SCAT in both OBS and GDM groups (P<0.01), and OMAT from GDM releases more glycerol than OMAT from NOBS and OBS women (P<0.01). In addition, we treated adipose tissues from NOBS women with different doses of ADM for 24 hours to assess the impact of ADM on glycerol release. ADM stimulated glycerol release from OMAT in a dose-dependent manner, and these increases were reduced by pre-incubation of OMAT with ADM antagonist, ADM22-52. In contrast, the SCAT did not show any response to the ADM treatment. These results suggested that ADM stimulates lipolysis in OMAT, but not SCAT, and the blockade of ADM with its antagonist can improve the lipid homeostasis in GDM.

## Discussion

Present study demonstrated that human SCAT and OMAT express mRNA for proinflammatory molecules, RBP4 and TLR-4, and adipogenic molecules, ChREBP and CEL; however no significant differences were noted between NOBS, OBS, and GDM women. On the other hand, the mRNA of MCP -1, CD68, and IL-6, IL-8, and TNF-α were significantly increased in OMAT, but not in SCAT, from GDM women compared to both OMAT and SCAT in NOBS and OBS women, and glucose stimulates human adipocyte MCP-1 mRNA expression. Both glucose and TNF-α dose-dependently stimulated ADM and its receptor components, CRLR, RAMP2 and RAMP3 mRNA in human adipocytes. ADM and its receptor components were significantly higher in OMAT from GDM women compared to all NOBS women. In both OBS and GDM women basal glycerol release was higher in OMAT compared to SCAT, and OMAT from GDM releases more glycerol than OMAT from NOBS and OBS subjects, and ADM dose-dependently stimulated glycerol release by OMAT, but not by SCAT in NOBS women. ADM-induced glycerol release was reduced by pre-incubation of AT with ADM22-52. Therefore, we propose that excessive ADM and its receptor expressions in OMAT, but not in SCAT appears to contribute to the lipid dysfunction in GDM women, and the increased proinflammatory molecules in OMAT may play a major role in stimulating ADM and its receptor expressions. Thus, present study on the role of ADM in adipose tissues may provide new insights into GDM pathophysiology and open new possibilities for its prevention and treatment.

It has been reported that maternal testosterone levels are associated with C-peptide levels in the Mexican American subset of the Hyperglycemia and Adverse Pregnancy Outcome (HAPO) Study cohort [26]. Hispanic women with polycystic ovarian syndrome have the most severe phenotype, both in terms of hyperandrogenism and metabolic criteria, and Non-Hispanic black women have an overall milder polycystic ovarian syndrome phenotype than Hispanics and non-Hispanic white women [27]. Present study showed a comparatively higher representation of Hispanic patients in the GDM group, raising the possibility that the differences in race/ethnic related sex hormone levels could potentially impacted the measured outcomes.

The association between obesity and insulin resistance has been previously reported [28]. However, since not all obese individuals are insulin resistant [29], fat mass per se, can’t be the sole determinant of insulin resistance. In the present study, the pre-pregnancy/early pregnancy BMI in OBS and GDM were significantly higher than NOBS group (P = 0.0006, Table 1), but there were no significant differences in BMI between OBS and GDM groups, suggesting that fat mass per se is not the sole determinant of insulin resistance in patients with GDM. In other words, metabolic dysregulation manifested in GDM may not be related to adipose tissue mass alone. Therefore, we examined the mRNA expression of some adipogenic and proinflammatory molecules to explore the mechanism by which adipose tissue may contribute to the dyslipidemia in GDM. Here we showed that mRNA expressions of RBP-4, TLR-4 and CEL did not exhibit any significant differences between SCAT and OMAT among NOBS, OBS and GDM groups (P>0.05, Fig 1). In the GDM subjects, only ChREBP mRNA expression was marginally but significantly higher in SCAT than that in OMAT. We propose that there might be small differences of adipokine expressions in SCAT and OMAT between groups that could not be detected in the current study due to the limited statistical power. However, the putative differences of such small alterations in adipokines may be biologically and pathophysiologically less significant compared to the regional differences in the altered lipid metabolism.

MCP-1 is a member of the adipokine family and promotes migration of inflammatory cells by chemotaxis and integrin activation [30], and to recruit monocytes from the blood into atherosclerotic lesions, thereby promoting foam cell formation [31]. We hypothesized that MCP-1 may serve as a signal that triggers inflammation by attracting macrophages into adipose tissues via secretion of a wide variety of inflammatory molecules [32], including TNF-α [33], interleukin-6 (IL-6) and IL-8 [34]. Our results showed that the mRNA of MCP-1 was substantially greater in OMAT from women with GDM when compared to non-GDM groups (Fig 2). In addition, macrophage marker CD68, and cytokines IL-6, IL-8, and TNF-α were also significantly increased in OMAT from GDM women compared to OMAT and SCAT in NOBS and OBS women (P<0.05). These findings are consistent with our previous study in GDM women showing increased macrophage infiltration [35] and imply a proinflammatory status in OMAT but not in SCAT in GDM women. Furthermore, the present study found that glucose stimulates MCP-1 mRNA levels in human adipocytes, this is in agreement with the literature showing that high glucose increased MCP-1 production in human vascular endothelial cells [36], indicating that hyperglycemia may play a major role in enhancing MCP-1 expression in OMAT and directly triggers the recruitment of macrophages to adipose tissues. The infiltrated macrophages may in turn secrete a variety of chemokines and other cytokines that further promote a local inflammatory response and affect gene expression in adipocytes, contributing to systemic insulin resistance.

Hyperglycemia itself in GDM may be one of the stimulants for the elevated ADM and its receptor expressions in adipocytes, thus contributing to the disturbed lipid metabolism by enhancing ADM’s action. Our results showed that glucose dose-dependently increased mRNA expressions of ADM and its receptor components CRLR, RAMP2 and RAMP3 (Fig 3A) suggesting the association between hyperglycemia and elevated ADM in diabetic patients. On the other hand, TNF-α is a cytokine largely expressed in adipose tissue in diabetes mellitus [33], which may be associated with impaired insulin sensitivity in GDM. Here we showed that TNF-α dose-dependently increased mRNA for ADM and its receptor components CRLR, RAMP2, and RAMP3 in the adipocytes (Fig 3B). Thus, we propose that increased circulating TNF-α level in GDM patients may further stimulate ADM and its receptor expressions in adipose tissue, and additively boost glucose actions on ADM system. In addition, similar actions of high glucose and TNF-α illustrated in Fig 3 may support the notion that increased expression of TNF-α is probably the consequence of hyperglycemia in GDM.

It has been reported that ADM concentrations in plasma from T2DM patients [19] and amniotic fluid from diabetic pregnancies are elevated compared to uncomplicated pregnancies [37], suggesting possible involvement of ADM in the pathogenesis of insulin resistance. We have previously demonstrated enhanced mRNA expression of ADM and its receptors in OMAT from GDM compared with normal controls [20], but the protein expression of ADM system and their reginal differences in pregnancy with NOBS, OBS, and GDM remain unknown. The present study showed that ADM and its receptor components CRLR, RAMP2 and RAMP3 were expressed in both SCAT and OMAT from NOBS, OBS, and GDM women (Figs 4 and 5). The intensity of ADM and CRLR, RAMP2 and RAMP3, were significantly higher in OMAT from GDM women compared to all other segments tested (P<0.05). However, no significant differences were observed in ADM and its receptor components between non-GDM groups, irrespective of the BMI, suggesting a consistent enhancement in both ADM and its receptors in adipose tissues from GDM patients, and the OMAT in OBS women did not exhibit the preferential ADM expression. Thus, we propose that ADM secreted by adipose tissue may alter the lipid metabolism through elevated receptors in paracrine manner.

The intra-abdominal fat stores are critical determinants of adipose-related metabolic complications [38]. Due to the anatomical location of visceral fat depots and their venous drainage by the portal vein system [39], intra-abdominal adipocytes are highly responsive to catecholamine stimulation and poorly responsive to lipolysis inhibition by insulin [40]. Therefore, OMAT may generate a high–FFA flux in the portal vein leading to increased triglyceride-rich lipoprotein in the liver and contribute to the development of metabolic complications [41]. The present study indicated that OMAT appeared to be substantially more responsive than SCAT to positive lipolytic stimuli ADM. This is the first study on the regional differences in adipocyte metabolism and comparing visceral versus subcutaneous parameters in pregnant women. The higher responsiveness of OMAT to positive lipolytic stimuli has also been found in other studies [42] and support the hypothesis that OMAT contribution is greater for the high–fatty acid flux to the liver, at least in physiological conditions of stimulated lipolysis.

GDM is accompanied by alterations in fasting and postprandial plasma concentrations of lipids, including increase in plasma triacylglycerol and FFAs, delayed postprandial clearance of fatty acids [43]. Increased circulating FFAs have also been associated with fetal overgrowth particularly of adipose tissue [44], suggesting that neonatal birth weight was positively correlated with concentrations of triacylglycerol and FFAs. By measuring the glycerol production, the breakdown product of triglycerides, present study demonstrated that basal glycerol release by OMAT was higher than SCAT in both OBS and GDM groups (Fig 6A), and OMAT from GDM women releases more glycerol than OMAT from NOBS and OBS subjects. Further, ADM stimulated glycerol release from OMAT into the culture medium in a dose-dependent manner, and these increases were reduced by pre-incubation with ADM22-52. In contrast, the SCAT did not show any response to the ADM treatment, suggesting that OMAT in GDM is more active in basal glycerol production, and the lipolytic effect of ADM was much more apparent in OMAT than in SCAT, and the blockade of ADM with its antagonist can improve the lipid homeostasis in GDM pregnancies. Limitations of this study include limited numbers of patients and the amount of collected tissues for additional measures of proteins and the activity of various regulatory enzymes.

Based upon our results, we conclude that increased proinflammatory molecules may play a major role in stimulating ADM and its receptor expressions in OMAT. Elevated ADM and its receptor in OMAT, but not in SCAT, appears to contribute to the lipid dysregulation in GDM women. Thus, ADM induces a state of lipolysis consistent with insulin resistance in GDM, and the alteration in lipid profile on the maternal side may affect the quantity and/or the quality of lipids being transferred to the fetus, thus leading to fetal macrosomia. Blockage of ADM’s actions maybe a new target of a therapeutic intervention for GDM.

## Supporting information

S1 File (DOCX)
