# Supplementary material for: Lipid dysfunction and adrenomedullin expression in omental versus subcutaneous adipose tissues in diabetic pregnancies
Source: PLoS One. 2022 Apr 7;17(4):e0265419. doi: 10.1371/journal.pone.0265419 (PMC8989323; doi:10.1371/journal.pone.0265419)
Supplement: S1 File — (DOCX) [file pone.0265419.s001.docx]

**Minimal Data Set for Manuscript of ADM in Human Adipose Tissue**

**Fig. 1. RBP-4 mRNA in human SCAT and OMAT**

| \|  \|  \| Mean \| SE \| \| --- \| --- \| --- \| --- \| \| NOBS \| SCAT \| 42.532855 \| 5.650211 \| \|  \| OMAT \| 41.599965 \| 5.801831 \| \| OBS \| SCAT \| 82.752075 \| 10.64942 \| \|  \| OMAT \| 60.964425 \| 19.31988 \| \| GDM \| SCAT \| 50.31316 \| 14.05152 \| \|  \| OMAT \| 66.56366833 \| 20.27664 \| |  |  |  |
| --- | --- | --- | --- | --- | --- | --- | --- | --- | --- | --- | --- | --- | --- | --- | --- | --- | --- | --- | --- | --- | --- | --- | --- | --- | --- | --- | --- | --- | --- | --- | --- |
|  |  |  |  |
|  |  |  |  |
| **TLR-4 mRNA**   \|  \|  \| Mean \| SE \| \| --- \| --- \| --- \| --- \| \| NOBS \| SCAT \| 32.69286267 \| 9.920785 \| \|  \| OMAT \| 36.01413667 \| 6.558075 \| \| OBS \| SCAT \| 20.239845 \| 3.351527 \| \|  \| OMAT \| 31.39751833 \| 7.800509 \| \| GDM \| SCAT \| 22.32291167 \| 3.872214 \| \|  \| OMAT \| 48.00246667 \| 6.224906 \| |  |  |  |
|  |  |  |  |
| **ChREBP mRNA**   \|  \|  \| Mean \| SE \| \| --- \| --- \| --- \| --- \| \| NOBS \| SCAT \| 9.692362833 \| 2.460203 \| \|  \| OMAT \| 9.183133 \| 1.129765 \| \| OBS \| SCAT \| 13.60490167 \| 1.688786 \| \|  \| OMAT \| 15.29281617 \| 2.521573 \| \| GDM \| SCAT \| 15.39913867 \| 1.896774 \| \|  \| OMAT \| 11.17623233 \| 1.274812 \| |  |  |  |
|  |  |  |  |

**CEL mRNA**

|  |  | Mean | SE |
| --- | --- | --- | --- |
| NOBS | SCAT | 13.74834083 | 3.011337 |
|  | OMAT | 17.49420167 | 5.773968 |
| OBS | SCAT | 9.840852833 | 1.425226 |
|  | OMAT | 12.94155433 | 2.085417 |
| GDM | SCAT | 15.88189033 | 3.006794 |
|  | OMAT | 17.8206535 | 4.021127 |

**Fig. 2. MCP-1 mRNA in human SCAT and OMAT**

|  |  | Mean | SE |
| --- | --- | --- | --- |
| NOBS | SCAT | 33.80853833 | 5.653804 |
|  | OMAT | 50.03234817 | 14.37265 |
| OBS | SCAT | 60.16744 | 5.720781 |
|  | OMAT | 99.24120667 | 7.859298 |
| GDM | SCAT | 75.69349167 | 10.12211 |
|  | OMAT | 230.4596333 | 34.82988 |

**CD68 mRNA**

|  |  | Mean | SE |
| --- | --- | --- | --- |
| NOBS | SCAT | 10.03667817 | 1.230503 |
|  | OMAT | 14.120224 | 2.034065 |
| OBS | SCAT | 16.95069333 | 1.126106 |
|  | OMAT | 16.24734 | 0.798344 |
| GDM | SCAT | 12.613686 | 1.508826 |
|  | OMAT | 28.499635 | 5.245281 |

**TNF-α mRNA**

|  |  | Mean | SE |
| --- | --- | --- | --- |
| NOBS | SCAT | 4.423373167 | 0.636449 |
|  | OMAT | 5.035268 | 0.52636 |
| OBS | SCAT | 6.613299 | 0.764322 |
|  | OMAT | 11.25981283 | 2.35732 |
| GDM | SCAT | 9.682181833 | 0.785487 |
|  | OMAT | 20.98393 | 4.129539 |

**IL-6 mRNA**

|  |  | Mean | SE |
| --- | --- | --- | --- |
| NOBS | SCAT | 12.895537 | 3.848778 |
|  | OMAT | 29.41981333 | 2.823323 |
| OBS | SCAT | 20.743895 | 1.495969 |
|  | OMAT | 78.993585 | 13.91488 |
| GDM | SCAT | 68.140195 | 2.866594 |
|  | OMAT | 146.4564933 | 13.54949 |

**IL-8 mRNA**

|  |  | Mean | SE |
| --- | --- | --- | --- |
| NOBS | SCAT | 9.1299115 | 0.850801 |
|  | OMAT | 8.796752167 | 1.130271 |
| OBS | SCAT | 9.885111167 | 1.498226 |
|  | OMAT | 15.09004833 | 0.939588 |
| GDM | SCAT | 23.46357 | 3.590706 |
|  | OMAT | 42.79043833 | 4.037046 |

**Glucose stimulates MCP-1 mRNA**

| Glucose | Mean | SE |
| --- | --- | --- |
| 1.5mg/ml | 1.94686 | 0.139955 |
| 2.0mg/ml | 6.463401 | 0.260919 |
| 2.5mg/ml | 4.273315 | 0.304552 |

| **Fig. 3A1 Effects of Glucose on ADM mRNA in Human Adipocytes** | | | | | | |
| --- | --- | --- | --- | --- | --- | --- |
| Glucose (mg/ml) | Mean | SE |  |  |  |  |
| 1.5 | 2.1914846 | 0.2774759 |  |  |  |  |
| 2 | 5.2187023 | 0.2758875 |  |  |  |  |
| 2.5 | 5.7676376 | 0.3946958 |  |  |  |  |
| 3 | 9.2557877 | 0.9274698 |  |  |  |  |
| 3.5 | 15.061679 | 1.7421263 |  |  |  |  |

| **Fig. 3A2 Effects of Glucose on CRLR mRNA** | | | | | | |
| --- | --- | --- | --- | --- | --- | --- |
| Glucose (mg/ml) | Mean | SE |  |  |  |  |
| 1.5 | 1.980247 | 0.178761 |  |  |  |  |
| 2 | 1.883364 | 0.221167 |  |  |  |  |
| 2.5 | 2.345946 | 0.184418 |  |  |  |  |
| 3 | 3.282465 | 0.325903 |  |  |  |  |
| 3.5 | 3.07533 | 0.236161 |  |  |  |  |

| **Fig. 3A3 Effects of Glucose on RAMP2 mRNA** | | | | | | |
| --- | --- | --- | --- | --- | --- | --- |
|  | Mean | SE |  |  |  |  |
| 1.5 | 1.818432889 | 0.188882 |  |  |  |  |
| 2 | 1.721448939 | 0.29263 |  |  |  |  |
| 2.5 | 2.117647475 | 0.285365 |  |  |  |  |
| 3 | 2.513592211 | 0.267362 |  |  |  |  |
| 3.5 | 4.756873942 | 0.317597 |  |  |  |  |

| **Fig. 3A4 Effects of Glucose on RAMP3 mRNA** | | | | | | | | | | | | | | |  |  |  |  |  |
| --- | --- | --- | --- | --- | --- | --- | --- | --- | --- | --- | --- | --- | --- | --- | --- | --- | --- | --- | --- |
| Glucose (mg/ml) | | | Mean | | SE | | |  |  |  | | |  | |  |  |  |  |  |
| 1.5 | | | 2.0666851 | | 0.4728823 | | |  |  |  | | |  | |  |  |  |  |  |
| 2 | | | 4.9914352 | | 1.013859 | | |  |  |  | | |  | |  |  |  |  |  |
| 2.5 | | | 5.7284742 | | 1.0819435 | | |  |  |  | | |  | |  |  |  |  |  |
| 3 | | | 6.1263181 | | 0.8161561 | | |  |  |  | | |  | |  |  |  |  |  |
| 3.5 | | | 15.33898 | | 3.5741322 | | |  |  |  | | |  | |  |  |  |  |  |
| **Fig. 3B1 Effects of TNF-α on ADM mRNA in Human Adipocytes** | | | | | | | | | | | | | | | | | | | |
| TNF-α | Mean | | | | SE | | | | |  | | | | | | |  | |  |
| 0 | 1.6718002 | | | | 0.241041 | | | | |  | | | | | | |  | |  |
| 0.01 | 5.4479912 | | | | 0.4924834 | | | | |  | | | | | | |  | |  |
| 0.05 | 7.3666752 | | | | 1.2906836 | | | | |  | | | | | | |  | |  |
| 0.1 | 8.720544 | | | | 0.7411343 | | | | |  | | | | | | |  | |  |
| 0.5 | 15.824444 | | | | 2.2300632 | | | | |  | | | | | | |  | |  |
| **Fig. 3B2 Effects of TNF-α on CRLR mRNA** | | | | | | | | | | | | | | | | | |  |  |
| TNF-α | | | Mean | | | SE | | | | |  | |  | |  |  | |  |  |
| 0 | | | 1.99828 | | | 0.238986 | | | | |  | |  | |  |  | |  |  |
| 0.01 | | | 2.594873 | | | 0.395791 | | | | |  | |  | |  |  | |  |  |
| 0.05 | | | 4.377433 | | | 0.621014 | | | | |  | |  | |  |  | |  |  |
| 0.1 | | | 6.444945 | | | 1.035871 | | | | |  | |  | |  |  | |  |  |
| 0.5 | | | 7.445183 | | | 1.012219 | | | | |  | |  | |  |  | |  |  |
|  | | |  | | |  | | | | |  | |  | |  |  | |  |  |

| **Fig. 3B3 Effects of TNF-α on RAMP2 mRNA** | | | | | | |
| --- | --- | --- | --- | --- | --- | --- |
| TNF-α | Mean | SE |  |  |  |  |
| 0 | 1.924983756 | 0.163329 |  |  |  |  |
| 0.01 | 1.981972637 | 0.34107 |  |  |  |  |
| 0.05 | 1.92574805 | 0.279576 |  |  |  |  |
| 0.1 | 2.052505532 | 0.213404 |  |  |  |  |
| 0.5 | 3.328857491 | 0.307883 |  |  |  |  |

| \| **Fig. 3B4 Effects of TNF-α on RAMP3 mRNA** \| \| \| \| \| \| \| \| --- \| --- \| --- \| --- \| --- \| --- \| --- \| \| TNF-α \| Mean \| SE \|  \|  \|  \|  \| \| 0 \| 3.2938752 \| 0.4909566 \|  \|  \|  \|  \| \| 0.01 \| 7.4227936 \| 2.0711345 \|  \|  \|  \|  \| \| 0.05 \| 11.086211 \| 1.9356287 \|  \|  \|  \|  \| \| 0.1 \| 13.60956 \| 2.7198292 \|  \|  \|  \|  \| \| 0.5 \| 16.986094 \| 3.7081193 \|  \|  \|  \|  \| |
| --- | --- | --- | --- | --- | --- | --- | --- | --- | --- | --- | --- | --- | --- | --- | --- | --- | --- | --- | --- | --- | --- | --- | --- | --- | --- | --- | --- | --- | --- | --- | --- | --- | --- | --- | --- | --- | --- | --- | --- | --- | --- | --- | --- | --- | --- | --- | --- | --- | --- |

**Fig. 4 Immunofluorescence of ADM in SCAT and OMAT**

|  |  | Mean | SE |
| --- | --- | --- | --- |
| NOBS | SCAT | 0.329809 | 0.060925 |
|  | OMAT | 1.42884 | 0.2285 |
| OBS | SCAT | 1.010971 | 0.312021 |
|  | OMAT | 1.819281 | 0.206556 |
| GDM | SCAT | 0.760058 | 0.136291 |
|  | OMAT | 2.852082 | 0.249762 |

**Fig. 4 Immunofluorescence of CRLR**

|  |  | Mean | SE |
| --- | --- | --- | --- |
| NOBS | SCAT | 0.793214 | 0.148579 |
|  | OMAT | 1.283735 | 0.264815 |
| OBS | SCAT | 1.138229 | 0.238372 |
|  | OMAT | 2.015436 | 0.410356 |
| GDM | SCAT | 1.960899 | 0.398087 |
|  | OMAT | 7.564901 | 0.782956 |

**Fig. 5 Immunofluorescence of RAMP2**

|  |  | Mean | SE |
| --- | --- | --- | --- |
| NOBS | SCAT | 0.663591 | 0.228796 |
|  | OMAT | 1.978912 | 0.401023 |
| OBS | SCAT | 0.803302 | 0.107628 |
|  | OMAT | 2.379242 | 0.258208 |
| GDM | SCAT | 1.39282 | 0.191736 |
|  | OMAT | 6.652432 | 1.12369 |

**Fig. 5 Immunofluorescence of RAMP3**

|  |  | Mean | SE |
| --- | --- | --- | --- |
| NOBS | SCAT | 1.057657 | 0.339751 |
|  | OMAT | 1.835736 | 0.291088 |
| OBS | SCAT | 1.147915 | 0.21289 |
|  | OMAT | 2.198347 | 0.453435 |
| GDM | SCAT | 1.882119 | 0.287311 |
|  | OMAT | 5.333945 | 0.520127 |

**Fig. 6 Basal Glycerol Release**

|  |  | Mean | SE |
| --- | --- | --- | --- |
| NOBS | SCAT | 5.79267 | 0.222738 |
|  | OMAT | 7.736586 | 0.128683 |
| OBS | SCAT | 8.853167 | 0.561041 |
|  | OMAT | 14.85565 | 0.644294 |
| GDM | SCAT | 11.10951 | 0.392464 |
|  | OMAT | 28.30552 | 1.976344 |

**Fig. 6 ADM-Stimulated Glycerol Release**

|  |  | Mean | SE |
| --- | --- | --- | --- |
| CTL | SCAT | 7.583295 | 0.29988 |
|  | OMAT | 10.51461 | 0.250569 |
| ADM1nM | SCAT | 9.932587 | 0.51119 |
|  | OMAT | 12.15271 | 0.459019 |
| ADM10nM | SCAT | 9.575131 | 0.600022 |
|  | OMAT | 18.52955 | 1.071573 |
| ADM100nM | SCAT | 12.30527 | 0.72509 |
|  | OMAT | 26.79803 | 1.270121 |
| ADM22-52 | SCAT | 8.875918 | 0.318282 |
|  | OMAT | 11.27122 | 0.977927 |
| ADM+ADM22-52 | SCAT | 8.029879 | 0.844639 |
|  | OMAT | 13.21178 | 0.403946 |
